# Feasibility of Assessing Economic and Sexual Risk Behaviors Using Text Message Surveys in African-American Young Adults Experiencing Homelessness and Unemployment: Single-Group Study

**DOI:** 10.2196/14833

**Published:** 2020-07-17

**Authors:** Larissa Jennings Mayo-Wilson, Nancy E Glass, Alain Labrique, Melissa Davoust, Fred M Ssewamala, Sebastian Linnemayr, Matthew W Johnson

**Affiliations:** 1 Department of Applied Health Science Indiana University School of Public Health Bloomington, IN United States; 2 Department of International Health Johns Hopkins University School of Public Health Baltimore, MD United States; 3 Johns Hopkins University School of Nursing Baltimore, MD United States; 4 Department of Health Law, Policy and Management Boston University School of Public Health Boston, MA United States; 5 The Brown School Washington University in St Louis St Louis, MO United States; 6 RAND Corporation Santa Monica, CA United States; 7 Behavioral Pharmacology Research John Hopkins University School of Medicine Baltimore, MD United States

**Keywords:** HIV, sexual risk behaviors, homelessness, text messages, young adults, economic, mobile phones

## Abstract

**Background:**

Text messages offer the potential to better evaluate HIV behavioral interventions using repeated longitudinal measures at a lower cost and research burden. However, they have been underused in US minority settings.

**Objective:**

This study aims to examine the feasibility of assessing economic and sexual risk behaviors using text message surveys.

**Methods:**

We conducted a single-group study with 17 African-American young adults, aged 18-24 years, who were economically disadvantaged and reported prior unprotected sex. Participants received a text message survey once each week for 5 weeks. The survey contained 14 questions with yes-no and numeric responses on sexual risk behaviors (ie, condomless sex, sex while high or drunk, and sex exchange) and economic behaviors (ie, income, employment, and money spent on HIV services or products). Feasibility measures were the number of participants who responded to the survey in a given week, the number of questions to which a participant responded in each survey, and the number of hours spent from sending a survey to participants to receiving their response in a given week. One discussion group was used to obtain feedback.

**Results:**

Overall, 65% (n=11/17) of the participants responded to at least one text message survey compared with 35% (n=6/17) of the participants who did not respond. The majority (n=7/11, 64%) of the responders were women. The majority (n=4/6, 67%) of nonresponders were men. An average of 7.6 participants (69%) responded in a given week. Response rates among ever responders ranged from 64% to 82% across the study period. The mean number of questions answered each week was 12.6 (SD 2.7; 90% of all questions), ranging from 72% to 100%. An average of 6.4 participants (84%) answered all 14 text message questions in a given week, ranging from 57% to 100%. Participants responded approximately 8.7 hours (SD 10.3) after receiving the survey. Participants were more likely to answer questions related to employment, condomless sex, and discussions with sex partners. Nonresponse or *skip* was more often used for questions at the end of the survey relating to sex exchange and money spent on HIV prevention services or products. Strengths of the text message survey were convenience, readability, short completion time, having repeated measures over time, and having incentives.

**Conclusions:**

Longitudinal text message surveys may be a valuable tool for assessing HIV-related economic and sexual risk behaviors.

**Trial Registration:**

ClinicalTrials.gov NCT03237871; https://clinicaltrials.gov/ct2/show/NCT03237871

## Introduction

Prior research has found that text messaging may be a promising strategy for involving young adults in research [1-7], as young adults are among the largest consumers of digital communication technologies [8-10]. More than 8 billion text messages are sent in the United States each day [8,11], and 97% of US young adults, aged 18 to 29 years, report using text messages at least once a day [8,12]. According to smartphone user data in the United States, young adults send and receive as many as 75 text messages per day, regardless of socioeconomic status [11].

In recent years, two-way text messages in the form of text message questionnaires (or surveys) have been used to obtain real-time data in health research settings [3,5,7,13-15]. Text messaging as an assessment tool has been valued given that it can be easily integrated into the lives of study participants, who often carry cell phones throughout the day, without being intrusive or requiring additional travel or study visitation time [16]. For researchers, data collection has a rapid turnaround time, is scalable to large groups, and is relatively inexpensive [3,4,16,17]. Participants may also be more responsive to the convenience of text messaging [16], and there is an additional benefit of anonymity when reporting sensitive behaviors, such as sexual activity, drug use, or housing instability [2]. In fact, one study found that text message responses from participants were more candid than responses from voice interviews [18]. Text message surveys yield data that are comparable with other paper and online assessment tools [19,20], while overcoming many of the limitations of these traditional approaches (ie, interviews, computer-assisted surveys, and school-based assessments) [4,7,21,22]. For example, real-time text message data can reduce recall biases inherent in costly assessments that may be several months or years apart [5,23]. More frequent text message surveys, which are administered daily or weekly over the life of a study, may also provide a more detailed picture of how behaviors change over time [1,5,23]. Obtaining data in real-time can also enable researchers to address any issues related to measurement or nonresponse promptly [24,25]. Text message surveys may also result in more representative research data by better engaging out-of-school individuals or individuals living in lower-income and underserved communities, who might otherwise be missed when using school- and clinic-based assessments [4,21].

Text message surveys have been used in many health areas, including diet and obesity [6,7], asthma [7], teen pregnancy [26], and depression [1]. However, with the exception of measuring medication adherence [3,5,24,25,27], two-way text message surveys have rarely been used in HIV prevention research. Sexual risk behaviors, such as unprotected sex, sex while intoxicated, or sex with multiple concurrent partners, are known to contribute to the spread of HIV [28-30]. As a result, reducing sexual risk-taking is a hallmark of many HIV behavioral prevention strategies, particularly among African American young adults who are disproportionately impacted by HIV [31-33]. Yet, despite the high rates of cell phone usage and the alarmingly high rates of HIV in African American young adults, few studies have used text messages to collect data on sexual behaviors [2]. Commonly used methods to collect data on sexual behaviors, such as those mentioned earlier (ie, interviews, computer-assisted surveys, and clinic visits), are less likely to measure behaviors in the most recent hours or days prior [5]. In addition, the economic drivers of HIV are rarely assessed using repeated measures. Prompting young adults to provide a text message reply regarding the frequency and type of sex they engaged in, in addition to other socioeconomic factors, may be a viable means of data collection, provided it is feasible, acceptable, and reliable.

The aim of this study was to examine the feasibility of assessing sexual and economic behaviors using text message surveys in African American young adults who were out-of-school and experiencing homelessness and unemployment in Baltimore, Maryland. The majority (82%) of HIV diagnoses in Baltimore is found among African Americans, with young adults, aged 20 to 29 years, representing the highest proportion [34]. Young adults in the city make up an increasing proportion of the homeless and unemployed [35,36]. Young adults experiencing homelessness are 6-12 times more likely to become infected with HIV than housed young adults, with prevalence rates as high as 12% [37-39]. HIV prevalence among African Americans in Baltimore is 3.1%, which is more than 10 times the national HIV prevalence in the United States (0.3%) and which exceeds the Joint United Nations Programme on HIV and AIDS’s definition of a generalized epidemic (HIV prevalence >1%) [34,40,41]. Specifically, this manuscript describes the process, challenges, and solutions regarding text message survey responsiveness and utility. In addition, it discusses the implications of using text message surveys in future HIV behavioral intervention trials.

## Methods

### Design

A single-group cohort study was used to examine the feasibility of assessing economic and sexual risk behaviors using weekly text message surveys. Participants were invited to respond to a text message survey sent to their cell phone every Monday at 9 AM for 5 weeks.

### Recruitment and Enrollment

Potential participants were recruited onsite from 2 community-based organizations (CBOs) providing emergency and supportive residential services to young adults in Baltimore, Maryland. A recruitment flyer was posted in the main building of both CBOs. Designated CBO staff introduced potential participants to the study team on scheduled visit days. Study eligibility was determined using a paper-based screening questionnaire that was administered by a trained research assistant. Individuals were eligible to participate if, at the time of enrollment, they were African American, aged 18-24 years, living in Baltimore, experiencing homelessness within the last 12 months (ie, defined as reporting any episode in which a person lacked a regular or adequate nighttime residence, such as a hotel/motel, vehicle, shelter, or friend’s home, and was living primarily on their own, apart from a parent or guardian), unemployed or underemployed (≤10 hours per week), out-of-school, reporting one or more episodes of unprotected sex in the last 12 months, and having a cell phone that could send and receive text messages. Eligible participants were then introduced to the study, and informed consent was obtained.

As part of the enrollment process, we invited participants to register their cell phone number with the text message survey app. Participants sent a text message with the word *join* to the study phone number to register. Each person then received a brief orientation regarding the survey’s content, timing, and payment incentive (US $20 in cash for answering 4 out of 5 weekly surveys). We also provided snacks and beverages. In the presence of a trained research assistant, the participant also completed a mock but identical version of the 14-question text message survey on his or her cell phone. This was done to confirm readability and understanding of the text message questions and prompts and to clarify any points of confusion. Participants were also advised on how to opt out of the survey by sending a text with the word *leave* at any time. As a final orientation step, participants were provided an informational sheet and advised on how to increase privacy during the study period and avoid unintended loss of confidentiality, such as activating cell phone passwords, deleting all text message surveys, responding only to the study’s phone number, and answering in a quiet and private space. Participants were also informed of the study’s security protocol that included separating cell phone numbers from identifying information; selecting a platform, such as TextIt.In, that did not require handing over participants’ names, addresses, or other identifying information to a mobile database software company; anonymizing phone numbers with a random code at the end of the study to render numbers invalid for future use; and using encrypted and compliant channels of TextIt.In.

### Text Survey Design

**Figure 1:**
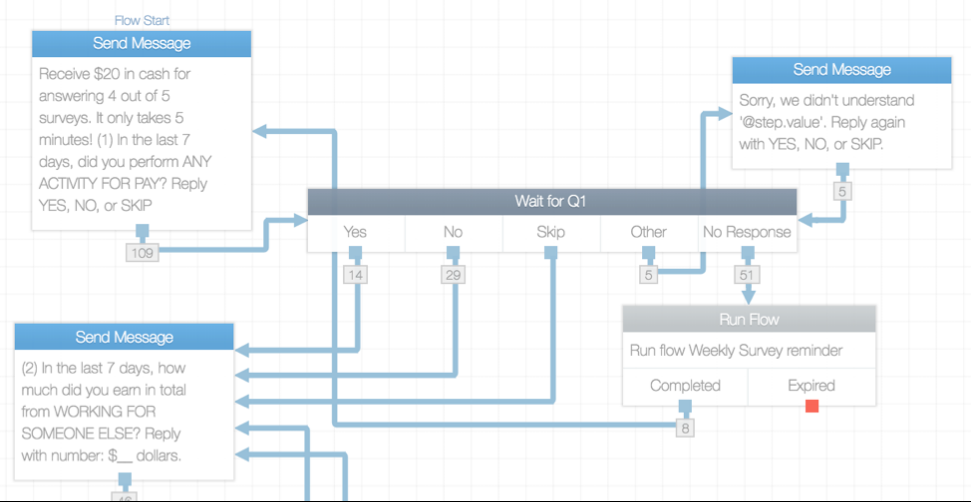
Screen shot of TextIt.In weekly survey flow (excerpt only).

We used *TextIt.In* to create, send, and receive text messages from participants. TextIt.In is an online service for building text messaging apps using a visual and interactive flow. The text message survey was powered by *Twilio*, a cloud communications platform, using a study-sponsored phone number. We then developed an online logic tree to order how survey questions would be texted to the participants. Figure 1 shows an excerpt of the branch logic used in the question tree. Each of the 14 questions was sent sequentially and in the same order as a single text message after the prior question had been answered. To facilitate responsiveness and data quality, the text messaging app included automated reminders and quality check prompts. Participants had 24 hours to complete each weekly text message survey. One automated text message reminder was sent to participants who did not initiate responding to the survey or to those who started but did not complete the survey within the first 24 hours. Reminder text messages included the name of the study, the payment incentive, and a reminder to respond to the survey within the next 24 hours. In addition, participants who responded with ineligible words or numbers outside of preset ranges received a text message query asking them to re-enter a valid response. All completed surveys generated 1 automatic text message that thanked the participant for his or her time.

### Measures

Data were collected in August and September 2017. Participants received the same 14 questions as text messages each week, regardless of their responses to the week’s prior text message survey. Table 1 lists the questions used in the survey of this formative study. The set of questions was adapted from previous studies of economic empowerment and costs associated with HIV preventive and treatment services [42-51] and included questions developed by the study team, specifically for African American young adults experiencing homelessness and unemployment [52-54]. All questions were in English and reviewed by the study team, piloted with young adults, and revised as necessary prior to utilization. The questions referred to the last 7 days, equivalent to the prior week. Eligible responses were yes/no or number of units (ie, dollars, episodes, and people). There were 7 economic questions relating to involvement in any type of paid work, the amount of cash earned from a job, the amount of cash earned from one’s own business, the amount of cash deposited into a savings account, the occurrence of loss of housing, the occurrence of requesting for cash to meet living expenses, and the amount of cash spent on any HIV preventive services or products (ie, condom or lubricant purchases, insurance copays for HIV testing or antiviral medications, and travel expenses to HIV educational sessions). An additional 7 sexual behavioral questions inquired about the number of sex partners, engagement in sex while high or drunk, frequency of condomless sex, utilization of other noncondom HIV preventive methods, frequency of sex exchange, discussion of HIV testing with sex partners, and receipt of HIV testing.

The primary feasibility measures of this study were: number of participants who responded to the survey in a given week, number of questions to which a participant responded in a given week, and number of hours from sending a survey to participants to receiving their response in a given week. We calculated the number, mean, and proportion of participants who responded to each question in each of the weekly surveys over the 5-week study period. A participant was categorized as responding to the question if he or she provided a valid response such as *yes/no*, a numerical response, a free-form text, or a *skip* response to proceed to the next question. A participant was categorized as responding to the survey if he or she provided a valid response to at least one question of the 14-question survey. Ever responders were defined as enrolled participants who responded to at least one text message survey over the course of the study period. Nonresponders were defined as enrolled participants who did not return a text message response to any of the text message surveys over the course of the study period. We considered the study to be feasible if we were able to identify and recruit >15 eligible participants within the study period. The study was also considered feasible if a mean question response rate of 70% or more among ever responders was achieved and if the mean response time was 24 hours or less. Feasibility was based on ever responders to account for any initial run-off of participants who signed up for the study but did not participate once the text message survey was initiated.

As part of the study’s process evaluation, 1 discussion group with 5 responders was used to obtain feedback. A recruitment flyer was posted in the main building of both CBOs, and all participants, including nonresponders, received a text message regarding the day, time, and location of the focus group discussion. The group discussion was moderated by the study PI who used a focus group discussion guide. Participants were asked to describe what they liked or disliked about the text message survey, what they considered to be barriers and facilitators to responding, and what changes they would recommend regarding the survey design (ie, questions, timing, and frequency), including suggestions for additional information or questions they would have liked to receive. Participant responses were documented using memo field notes that were expanded upon immediately after the discussion.

**Table 1:** List of questions included in the weekly text message survey with response options.

Indicator	Text message question	Response options^a^
Employment	In the last 7 days, did you perform any activity for pay?	01 Yes; 02 No
Income earned from job	In the last 7 days, how much did you earn in total from working for someone else?	US $
Income earned from own business	In the last 7 days, how much did you earn from being self-employed or from your own business?	US $
Savings	In the last 7 days, how much did you deposit into a savings account?	US $
Housing stability	In the last 7 days, have you been without a place to stay?	01 Yes; 02 No
Financial distress	In the last 7 days, did you ask someone for money to meet your food, housing, or other living expenses?	01 Yes; 02 No
Money spent on HIV prevention	How much have you spent in the last 7 days on HIV prevention?	US $
Sex partners	In the last 7 days, how many people have you had sex with?	__ # people
Sex while drunk or high	With any of these people, were you drunk or high while having sex?	01 Yes; 02 No
Condomless sex	In the last 7 days, how many times did you have sex without a condom?	__ # of times
Noncondom HIV preventive methods	Not including a condom, what other method(s) did you use to prevent HIV in the last 7 days?	Free text
Sex exchange	In the last 7 days, how many times did you receive money, food, or drugs in exchange for having sex?	__ # of times
Discussion of HIV testing	In the last 7 days, did you discuss HIV testing with your sex partner(s)?	01 Yes; 02 No
Uptake of HIV testing	In the last 7 days, did you get tested for HIV?	01 Yes; 02 No

^a^All questions included a response option of *skip*.

### Analysis

To analyze the results of the text message survey, we first created a database in Excel that included: a cell phone number for each participant; a participant unique study ID; demographic data relating to participants’ age, gender, education level, years living in Baltimore, number of hours worked per week, and number of children living in and out of the household, the date and time of study enrollment, the date and time of all outgoing and/or incoming messages, and the numerical, textual, or free-from text message response to each of the 14 text message questions each week. Secondly, we calculated the study’s primary feasibility measures, as listed above. Third, we calculated the frequencies of sexual and economic behaviors per the specific responses for each weekly question. Finally, lessons learned from the study’s process evaluation were analyzed over 5 implementation domains: acceptability, enrollment and registration, responsiveness, data quality, and data access. This process involved a close reading of the study’s field notes, coding lessons learned by each implementation domain, and discussing findings with the study team.

### Ethics Approval

This study received ethics approval from the Johns Hopkins Bloomberg School of Public Health institutional review board (IRB#00007563).

### Availability of Data and Materials

The dataset analyzed during this study is available in the Mendeley repository [55].

## Results

### Sample Characteristics

A total of 17 participants were enrolled in the study, accounting for 1 of the study’s 3 feasibility criteria. Table 2 describes the sample’s demographic characteristics. All participants (n=17/17, 100%) were African American (per inclusion criteria), living in Baltimore, and recruited from 1 of the 2 community organizations providing support to young adults experiencing homelessness. The mean age was 21.2 years (SD 2.1). 53% (n=9/17) of participants were female and 47% (n=8/17) of the participants were male. About half of participants (n=9/17, 53%) had not received a high school diploma or equivalent. None were currently enrolled in school. The majority of participants were unemployed (n=13/17, 76%), and 24% (n=4/17) were working part-time. 29% (n=5/17) were parents with children living in or outside of their household.

**Table 2:** Demographic characteristics of participants.

Sample characteristics		Response group				Total	
		Ever responders		Nonresponders			
Number of participants, n (%)		11 (65%)		6 (35%)		17 (100%)	
Age (years), mean (SD)		21.0 (1.9)		21.5 (2.5)		21.2 (2.1)	
**African American^a^, n (%)**							
	Yes		11 (100)		6 (100)		17 (100)
	No		0 (0)		0 (0)		0 (0)
**Gender, n (%)**							
	Male		4 (36)		4 (67)		8 (47)
	Female		7 (64)		2 (33)		9 (53)
**Recruited from** **community-based organizations for homeless young adults^a^, n (%)**							
	Yes		11 (100)		6 (100)		17 (100)
	No		0 (0)		0 (0)		0 (0)
**Highest level of education, n (%)**							
	<12th grade		5 (45)		4 (67)		9 (53)
	High school diploma or equivalent		6 (55)		2 (33)		8 (47)
	Post-baccalaureate		0 (0)		0 (0)		0 (0)
**Currently enrolled in school^a^, n (%)**							
	Yes		0 (0)		0 (0)		0 (0)
	No		11 (100)		6 (100)		17 (100)
**Employment status^a^, n (%)**							
	Unemployed		8 (73)		5 (83)		13 (76)
	Employed part-time		3 (27)		1 (17)		4 (24)
	Employed full-time		0 (0)		0 (0)		0 (0)
Number of years living in Baltimore, mean (SD)		17.5 (6.1)		21.5 (2.5)		18.9 (5.4)	
**Are parents, n (%)**							
	Yes		4 (36)		1 (17)		5 (29)
	No		7 (64)		5 (83)		12 (71)

^a^Per inclusion criteria.

### Text Survey Responsiveness

Table 3 describes additional feasibility measures of the study. 65% (n=11/17) of participants responded to at least 1 text message survey over the 5-week study period compared with 35% (n=6/17) of participants who never responded. The majority (n=7/11, 64%) of ever responders were young women. The majority (n=4/6, 67%) of never responders were young men (Table 2). Among those who ever responded, an average of 7.6 participants responded to the text message survey in any given week (69% response rate; Table 3). Response rates among ever responders ranged from 64% to 82% across the 5-week study period, representing 62.7% of all survey questions in all 5 weeks. When participants responded in a given week, they also answered the majority of the 14 survey questions. The mean number of answered questions for responders in a given week was 12.6 (SD 2.7; 90% of all questions), ranging from 72% to 100% of all questions. This met the study’s feasibility criteria of an average weekly question response rate of 70% or more among ever responders. An average of 6.4 participants (84%) answered all 14 text message survey questions in a given week, ranging from 57% to 100%. Participants responded on average 8.7 hours (SD 10.3) after receiving the survey. In week 1, participants responded the fastest with an average of 1.7 hours (SD 2.2). The slowest mean time to response was 12.6 hours (SD 13.2) in week 3. This met the study’s feasibility criteria of a mean response time of <24 hours.

Table 4 presents the number of responders per question per week among ever-responding participants. The questions at the beginning of the survey had the highest response rates. Response rates were comparable across all questions in weeks 2 and 5 but tapered at the end of the survey in weeks 1, 3, and 4. In week 5, 1 participant answered the first question of the survey but omitted answering any further questions. Participants were most responsive to questions about employment, condomless sex, and discussions with sex partners. Nonresponse was highest for questions relating to sex exchange and money spent on HIV prevention products or services. The text messaging app successfully sent and received 1289 text messages with few errors (0.1%), indicating relative efficiency and reliability (Table 3).

**Table 3:** Feasibility measures among all and ever-responding participants by week and in total.

Study subgroups		Week						Total
		1	2	3	4	5		
**All participants (n=17), n**								
	Number of weekly text message surveys sent out	17	17	17	17	17	85	
	Number of text messages sent and received (includes all welcome, survey, reminder, correction, and thank you messages)	197	291	298	236	267	1289	
**Ever-responding participants (n=11)**								
	Participants^a^ who responded to the survey each week, n (%)	7 (64)	8 (72)	9 (82)	7 (64)	7 (64)	7.6 (69)	
	Questions participants^a^ responded to each week, mean (SD) (% out of 14)	10.1, 5.2 (72)	14.0, 0.0 (100)	13.6, 1.3 (97)	13.3, 1.9 (95)	12.1, 4.9 (86)	12.6, 2.7 (90)	
	Hours from sending survey to receiving participants’^a^ response each week, mean (SD)	1.7 (2.2)	10.2 (12.2)	12.6 (13.2)	4.9 (8.6)	13.9 (15.4)	8.7 (10.3)	
	Participants^a,b^ who responded to all 14 questions each week, n (%)	4 (57)	8 (100)	8 (89)	6 (86)	6 (86)	6.4 (84)	

^a^Never responders are excluded.

^b^Nonresponders for the specific week are excluded.

**Table 4:** Number of responders per question per week.

Question number	Week				
	1, n	2, n	3, n	4, n	5, n
1	7	8	9	7	7
2	7	8	9	7	6
3	6	8	9	7	6
4	6	8	9	7	6
5	6	8	9	7	6
6	6	8	9	7	6
7	5	8	9	7	6
8	5	8	9	7	6
9	5	8	9	7	6
10	4	8	9	6	6
11	4	8	8	6	6
12	4	8	8	6	6
13	4	8	8	6	6
14	4	8	8	6	6

### Reported Economic and Sexual Behaviors

Weekly economic and sexual behaviors reported by the participants are shown in Table 5. Employment rates remained low, ranging from 14% to 43% over the study period. Mean earnings from employment by others or from the participant’s own business ranged from US $37 (SD 62.8) to US $146 (SD 220.3) per week and from US $7 (SD 18.9) to US $55 (SD 107.2) per week, respectively (Table 5). Participants experiencing housing instability decreased from 43% to 0% over the course of the study period, as did the proportion of those requesting money from others to cover living expenses (57%-0%). For most weeks, no money was spent on HIV prevention services or products, such as condoms, HIV testing, lubricants, or antiviral medications. For 3 of the 5 weeks, approximately 14% of participants reported having sex while high or drunk at least once in the past week. Condomless sex was a common risk behavior, with 14%-75% of participants reporting condomless sex at least once in a given week. There were no reports of sex exchange for money, food, or housing. Using other noncondom prevention methods was also low (11% in week 3). Participants were more likely to respond *yes* to the last two sexual behaviors of the survey, which were about discussing HIV testing with any of their sex partners (43%-67%) and receiving an HIV test in the past week (14%-43%). Participants used the *skip* response infrequently and only during weeks 1 and 2. When used, *skip* was most common for questions relating to sex exchange and money spent on HIV prevention services or products (Table 5). The dataset analyzed during this study is publicly available [55].

**Table 5:** Reported economic and sexual behaviors in the last 7 days by ever responders.

Question number	Indicator	Number of times *skip* response was used	Week				
			1	2	3	4	5
1	Participants who performed any activity for pay, %	0	14	38	33	43	43
2	Earnings from job (US $), mean (SD)	1	69.7 (94.5)	145.6 (220.3)	114.4 (141.9)	36.6 (62.8)	85.7 (127.7)
3	Earnings from self-employment or own business (US $), mean (SD)	0	54.5 (107.2)	18.6 (49.1)	34.4 (48.8)	7.1 (18.9)	16.7 (40.8)
4	Reported savings in (US $), mean (SD)	1	48.6 (66.6)	20.5 (35.2)	28.9 (39.8)	18.6 (32.9)	18.3 (38.0)
5	Participants reporting having no place to stay, %	0	43	25	22	14	0
6	Participants who asked for money for living expenses, %	0	57	38	33	14	0
7	Reported spending on HIV prevention in (US $), mean (SD)	2	0 (0.0)	51.4 (136.1)	25.6 (66.2)	0 (0.0)	0 (0.0)
8	Number of sex partners in past week, mean (SD)	0	1.0 (0.7)	1.4 (0.7)	0.8 (0.7)	0.6 (0.5)	0.5 (0.5)
9	Participants who were drunk or high while having sex (at least once), %	0	14	13	0	14	0
10	Participants who reported condomless sex at least once in the past week, %	0	43	75	33	29	14
11	Participants who reported noncondom prevention methods, %	0	0	0	11	0	0
12	Participants who reported sex exchange in the past week, %	2	0	0	0	0	0
13	Participants who discussed HIV testing with sex partners, %	1	57	50	67	71	43
14	Participants who received an HIV test, %	1	14	25	22	14	43

### Implementation Lessons Learned

Table 6 summarizes the successes, challenges, and lessons learned in using text message surveys in this population. Key successes included participant acceptability, willingness to respond to the survey, confirming readability and functionality using a mock text message survey at enrollment, having moderately high responsiveness, and building in quality checks. Implementation challenges were low responses to questions perceived as sensitive or stigmatizing, technological delays, and the time required for restructuring text message data for analysis. Additional feedback from responders in a post-study discussion was that having the text message surveys arrive weekly and at the same time was helpful, as participants were always on their cell phones and available to respond quickly and conveniently. This, along with receiving cash payments, was viewed as a positive outcome. However, the reported weaknesses were that, for some, receiving the same set of questions each week was repetitive and may have contributed to response fatigue. Participants also requested whether informational text messages such as job announcements or sexual health tips could be provided as a reward for responding to each week’s survey. The study team’s observations while implementing the text message survey was that reducing text message wording, including using response prompts (eg, *reply with*: yes/no, US $ dollars, and # of times), and reminders were important to facilitating participation.

**Table 6:** Summary of text message surveys’ successes, challenges, and lessons learned by implementation domain.

Implementation domain	Successes	Challenges	Lessons learned
Acceptability	Participants were eager to enroll and motivated by cash payments. Willingness to respond to sensitive questions was enhanced by privacy supports.	Response declined at the end of the survey. The reasons for nonresponse are not well known because of lost to follow-up.	Participants valued text message contact but requested to receive nonrepeated survey questions and texts on jobs or sexual health.
Enrollment and registration	Readability and function of the survey were confirmed at enrollment for all participants who answered a mock survey and clarified points of confusion.	Some interested young adults did not have a working cell phone. Long wording of some questions appeared as multiple texts on small screens during enrollment.	Financial support for accessing cellular service may be needed to enroll more disconnected young adults.
Responsiveness	Two-thirds of participants responded to the survey representing a moderately high response rate. No participants used the opt-out function.	One-third of participants (mostly men) enrolled but never responded. One participant responded to only the first question.	Increasing incentives, reducing the number of questions, or reducing the frequency of surveys may improve responsiveness.
Data quality	A 7-day window and sending surveys on the same day and time were used to reduce recall bias. Query text messages were sent for invalid responses.	All data were self-reported and not administered by a researcher. The recall period of later responders may have included overlapping days.	More efforts are needed to assess data quality in lieu of response prompts, larger sample size, and responses over time.
Data access	Data were available at low cost and in real time at the moment when the participant responded.	All output was generated into separate weekly files that required time-consuming restructuring.	Routinely restructuring data would facilitate real-time analysis of individual and aggregate statistics.

## Discussion

### Principal Findings

The goal of this study was to examine the feasibility of a relatively new mode of data collection using text message surveys in a high HIV prevalence urban and ethnic minority setting. We found that using weekly automated text message surveys with short assessments was feasible with vulnerable young adults. Data collection with this population can be challenging, given the unpredictability of young adults’ schedules and the uncertainty regarding their interests in research participation. However, the majority of invited participants completed the survey and were receptive to answering the study’s text message questions. To our knowledge, this is one of the first studies in an urban setting to use text message surveys to assess economic and sexual risk behaviors in economically-vulnerable African American young adults, who also had little experience responding to text messages for research purposes.

The study’s experience is informative with regard to 3 research areas: acceptability of text message surveys, survey responsiveness, and implications for future studies relating to efficiency and data quality. First, in the context of acceptability, several factors may have contributed to the study’s generally positive reception. This study’s recruitment process began with an introduction to the study to young adults in the presence of their peers at the CBO center. Therefore, potential participants had an opportunity to enjoy snacks, ask questions, and determine their own interests, including the interests of their peers, in participating in the study. The study team also explained the cash incentives being used to compensate individuals who completed the 5-week test cycle. Although eligible young adults appeared to be motivated by cash payments, other potential drivers to participation may have been the perceived benefit of participating in a study on HIV prevention with friends, including being prompted to think about or discuss HIV. Another driver to participation may have been interests in using text messages as a new means of income generation, since nearly all young adults had a cell phone and were underemployed. The study also invited each participant to try a mock text message survey on their cell phone in a private location at the CBO. This enabled them to see what they would be receiving each week and to confirm their capacity to respond. It was our experience that participants found responding to relatively sensitive text message questions on economic and sexual behaviors acceptable, given the readability of the questions, the short completion time required (about 3 to 4 minutes), the anonymity of their cell phone, the ability to use phone passwords for additional privacy, and the option to delete all text messages.

A second area of consideration is responsiveness to the text message survey. Among all participants, the response rate of 65% was moderately high, representing nearly two-thirds of participants. For those individuals who responded to at least one text message survey, response rates were even higher in a given week, ranging from 64% to 82%, with participants answering about 90% of all questions. This study’s findings included higher response rates than similar text message survey studies, including 3 studies assessing substance use and sexual risk behaviors with US young adults aged 18 to 25 years (49% response rate) [2], medication adherence in HIV-negative transgender men and women (39% response rate) [3], and medication adherence from caregivers of HIV-infected children in Uganda (24% response rate) [56]. On the other hand, we have similar response rates as 2 additional studies assessing drug adherence using text message surveys with lesbian, gay, bisexual, and transgender young adults in the United States living with HIV (61% response rate) [5] and assessing quality of life via text message surveys with older patients with rheumatoid arthritis (69% response rate) [14]. Although higher responsiveness may be needed if text messages are the sole form of evaluation, such engagement by predominantly financially- and residentially-unstable young adults is encouraging. In addition, the average time to response ranged from 2 to 12 hours, representing a relatively rapid response period compared with mail-in or online surveys that may experience several days or weeks between distribution and response. Potential contributing factors may have been that the study’s in-person orientation process allowed participants to feel prepared in knowing what type of questions would be asked and how long it would take to respond. Our sequentially sending each question only after the prior question had been answered further allowed participants to track and respond to questions at their own pace. Being female also appeared to aid responsiveness, although more research is needed to understand whether and why this may be the case.

To that end, it is important to consider the nonresponsiveness observed in this study. The reasons for nonresponse may have been poor network coverage, having one’s cell phone lost/stolen, or lacking sufficient charge or cellular credit. The potential to earn cash payments spurred interest in many participants. However, for some, this enthusiasm may have waned over time. Nonresponsiveness may also have resulted from experiencing negative emotions when thinking about financial hardships or prior sex partners. It is possible that some participants simply wanted a break from the study but chose not to use the opt-out commands (*leave* or *stop*). Future studies should assess reasons for nonresponse, as this could increase the number of participants providing study data. Increasing incentives, reducing the number of questions, or reducing the frequency of text message surveys may also improve responsiveness. Paying participants more frequently rather than at the end of the study and requesting alternative forms of contact (ie, email and social media) to reconnect with nonresponders may additionally be helpful. Greater engagement might also be achieved if the text message surveys are concurrently embedded within an intervention or other in-person contact targeting the outcomes of the text assessment.

A final important area relates to implications for future research. Our text message surveys showed some promise as a measurement tool in behavioral research. Having repeated measures each week from participants provides stronger statistical power and enables trialists to better characterize fluctuations over time. Our text message survey was configured to query again any invalid or out-of-range responses to maximize data quality over time. Once the survey flow was automated and launched, there was minimal maintenance. However, despite having data available from the moment participants responded, the process of restructuring and aggregating weekly data files was time-consuming and resulted in the team generally viewing and analyzing data at the end of the study, rather than on a weekly basis. Improvements in exporting and coding the messaging app’s data would increase efficiency. Finally, given that text messaging technology is constantly evolving as are young adults’ cell phone behaviors, including the option to respond to surveys via instant messaging or other text messaging apps may improve participation.

The study’s small sample size was a limiting factor, as the study was not designed to determine efficacy or estimate prevalence of economic and sexual risk behaviors. Rather, the study aimed to conduct a rapid test cycle using a small group of young adults with a preliminary goal of assessing instrument feasibility for a larger intervention trial. We additionally selected young adults who had a working cell phone, were literate, and were receiving residential services at the participating CBOs. Although the participants were vulnerable in other ways, such a sampling strategy could mean that the findings are not generalizable to more disconnected young adults. In addition, although not using an interviewer to administer surveys may have increased participants’ responsiveness to sensitive questions, the use of self-administered assessments could have reduced data quality. Finally, although weekly assessments provided more frequent contact than traditional pre-post study designs, asking participants to recall economic and sexual behaviors in the last 7 days rather than the day before via daily surveys may have been challenging for some participants. Despite these limitations, the study was successful in monitoring behaviors over time. This study’s findings provide support for using text message surveys to collect data in future behavioral trials.

### Conclusions

Text messages offer the potential to better evaluate HIV behavioral interventions using repeated longitudinal measures at lower cost and research burden. However, they have been underutilized in US minority settings. We found that using weekly automated text message surveys with short assessments was feasible with vulnerable young adults. Additional research should focus on maintaining high responsiveness, improving the efficiency of data analysis, and ensuring data quality.

## Abbreviations

CBO: community-based organization
HIV: human immunodeficiency virus
